# Effect of the application of cattle urine with or without the nitrification inhibitor DCD, and dung on greenhouse gas emissions from a UK grassland soil

**DOI:** 10.1016/j.agee.2016.10.025

**Published:** 2016-11-01

**Authors:** L.M. Cardenas, T.M. Misselbrook, C. Hodgson, N. Donovan, S. Gilhespy, K.A. Smith, M.S. Dhanoa, D. Chadwick

**Affiliations:** a Rothamsted Research, North Wyke, Okehampton EX20 2SB, UK; b School of Geosciences, University of Edinburgh, Crew Building, Alexander Crum Brown Road, Edinburgh EH9 3FF, UK; c Woodlands One, Pomeroy Villas, Totnes, Devon TQ9 5BE, UK; d School of Environment, Natural Resources and Geography, Bangor University, Deiniol Rd., Bangor LL57 2UW, UK

**Keywords:** Nitrous oxide, Urine, Dung, Dicyandiamide, DCD, Meta-analysis

## Abstract

Emissions of nitrous oxide (N_2_O) from soils from grazed grasslands have large uncertainty due to the great spatial variability of excreta deposition, resulting in heterogeneous distribution of nutrients. The contribution of urine to the labile N pool, much larger than that from dung, is likely to be a major source of emissions so efforts to determine N_2_O emission factors (EFs) from urine and dung deposition are required to improve the inventory of greenhouse gases from agriculture. We investigated the effect of the application of cattle urine and dung at different times of the grazing season on N_2_O emissions from a grassland clay loam soil. Methane emissions were also quantified. We assessed the effect of a nitrification inhibitor, dicyandiamide (DCD), on N_2_O emissions from urine application and also included an artificial urine treatment. There were significant differences in N_2_O EFs between treatments in the spring (largest from urine and lowest from dung) but not in the summer and autumn applications. We also found that there was a significant effect of season (largest in spring) but not of treatment on the N_2_O EFs. The resulting EF values were 2.96, 0.56 and 0.11% of applied N for urine for spring, summer and autumn applications, respectively. The N_2_O EF values for dung were 0.14, 0.39 and 0.10% for spring, summer and autumn applications, respectively. The inhibitor was effective in reducing N_2_O emissions for the spring application only. Methane emissions were larger from the dung application but there were no significant differences between treatments across season of application.

## Highlights

• N_2_O EFs from urine deposition to grassland are larger if applied in spring.
• Meta-analysis showed a significant effect of season and not of treatment on the N_2_O EFs.
• Methane emissions were larger from the dung application compared to urine.
• CH_4_ totals were significantly different across seasons (lowest in spring).
• CH_4_ totals were not significantly different between treatments.

## 1 Introduction

For estimating nitrous oxide (N_2_O) emissions from soils, the current UK greenhouse gas (GHG) inventory is predominantly calculated using the emission factors (EFs) provided by the standard Tier 1 methodology of the Intergovernmental Panel on Climate Change (IPCC, 2006), because of the lack of country-specific data. The default emission factor (EF) for N_2_O emissions from cattle excreta deposited during grazing is 2% (IPCC, 2006). However, EF values for urine on grazed pasture for 5 UK studies summarised by van Groenigen et al. (2005a) showed large variability, ranging between 0.07% and 15.5%.

In grassland grazed by livestock, gaseous emissions from the returns of nitrogen (N) and carbon (C) in excreta differ between urine and dung. Urine, due to its high available N content and water deposited on the soil in each urination event has a large potential for emissions, especially when it infiltrates the soil profile. Emissions of N_2_O can occur from nitrification of the ammonium (NH_4_^+^) formed from the hydrolysis of urea in the urine, and/or from denitrification of the nitrite (NO_2_^−^) and nitrate (NO_3_^−^) resulting from nitrification of the ammonium (Kool et al., 2006b); the dominant process will depend on the environmental conditions. Dung, on the other hand, is rich in C in addition to N (mainly organic), providing energy for respiration; wet dung pats lying on the soil surface may inhibit aeration and promote anaerobicity in the pat and adjacent soil (Groenigen et al., 2005), stimulating N_2_O emission via denitrification and possibly also the release of methane (CH_4_). Emissions of both N_2_O and CH_4_ are influenced by environmental factors, especially rainfall and temperature, so the time of the year of urine and dung deposition is very relevant for a potential seasonal effect of grazing.

Urine patches represent a large source of direct and indirect N_2_O emissions, but it has been suggested by Kool et al. (2006a) and van Groenigen et al. (2005a) that secondary urine metabolites, e.g. hippuric acid, reduce N_2_O production by production of benzoic acid, an inhibitor of microbial activity (Fenner et al., 2005). However, other studies (Clough et al., 2009, Krol et al., 2015) reported no effect of hippuric acid on emissions and it is thought the difference could have been due to soil moisture, pH, or to the fact that the plant was excluded in earlier experiments. Synthetic nitrification inhibitors such as dicyandiamide (DCD) inhibit N_2_O emissions (Scheer et al., 2014) but their effectiveness in the field is variable, due to environmental factors, including soil temperature (Di and Cameron, 2002), soil texture (Bronson et al., 1989, McGeough et al., 2016) and rainfall (Shepherd et al., 2014). Recent findings report an effect of soil type potentially related to the organic matter content and microbial activity (Cahalan et al., 2015, McGeough et al., 2016).

In summary, because of the complexity of the emission processes, the default EF for N_2_O from cattle excreta is likely to misrepresent the true N_2_O emission rate. Therefore, efforts to reduce the uncertainty in this EF are needed to improve the accuracy of the UK inventory (Skiba et al., 2012). The UK’s Greenhouse Gas Platform Programme (www.greenhousegasplatformprogramme.org) was a national UK project which was undertaken partly for this purpose, and to help to assess the effectiveness of mitigation strategies. Grazed land can also act as a source or sink of atmospheric methane (CH_4_) (Yamulki et al., 1999), depending on the activity of soil methanogens and methanotrophs, which are affected by environmental conditions. Assessment of the total greenhouse balance is regarded as providing useful information on the true impact of this land use on climate change.

In this study we investigated the effect of the application of cattle urine and dung at different times of the grazing season on N_2_O and CH_4_ emissions on a UK grassland site. A control treatment receiving no dung or urine application was included, and artificial urine was also applied as a reference treatment. An additional treatment included a nitrification inhibitor, DCD, that was applied with the urine to assess the N_2_O emission mitigation potential of DCD in this soil. Differences in herbage dry matter production and soil mineral nitrogen dynamics between the treatments were also assessed.

## 2 Materials and methods

### 2.1 Site description

**Fig. 1:**
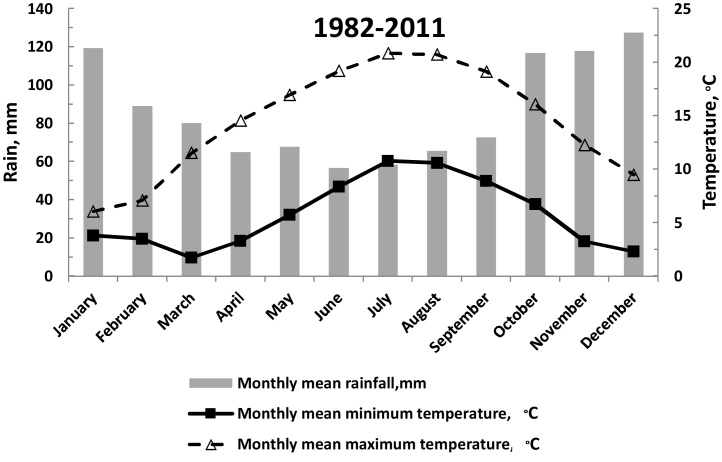
Long term average temperature and rainfall [1982–2011].

Experiments were conducted at the Rothamsted Research North Wyke farm, Devon, UK (50°45′N, 3°50′W), on a permanent grassland field between March 2012 and September 2013. The site was previously used for silage production, with no grazing livestock, for 3 years prior to the start of experiment. The average annual air temperature of the site is 9.6 °C, and the average annual precipitation is 1055 mm (30-year mean, climate record of North Wyke 1982–2012). Monthly rainfall is lowest in the summer, at which time temperatures are at their highest (Fig. 1). The soil type is a poorly drained silty clay loam (Halstow Series); (Harrod and Hogan, 2008). Previously developed protocols were used to guide on sampling strategies and management of the plots (Bell et al., 2015b).

Initial soil characterisation was carried out by measuring pH (in water); extractable phosphorus (P) as Olsen P; potassium (K) and magnesium (Mg) by extracting with ammonium nitrate solution followed by determination by flame photometry; sulphur (S) using a phosphate buffer extracting solution with ratio 1:2 and determination by Inductively Coupled Plasma Emission Spectroscopy; total N was determined by a thermal conductivity detector after sample combustion; total organic C by modified Walkley Black (Walkley and Black, 1934) and loss on ignition (LOI) methods; particle size distribution (PSD) – sand, silt, clay using a laser diffraction particle sizer; available water capacity (AWC) and bulk density (BD) (Table 1). The AWC was measured by assessing the soil moisture release characteristics using a sand bath and ceramic pressure plates for field capacity and permanent wilting point determination, respectively.

### 2.2 Treatments applied

Treatments were applied on 15th May, 3rd July and 26th September 2012, for the spring, summer and autumn experiments, respectively. The aim of these timings was to reflect deposition at different stages of the grazing season (early, mid and late). New plots were established for each application timing. The treatments applied were: natural urine (NU), natural urine + DCD (NU + DCD), artificial urine (AU), dung (D) and a control (C) that had nothing applied to the soil (see Table 2). The natural urine and dung were collected from Holstein dairy cows at Reading University, UK, stored in sealed vessels at <4 °C and not frozen before application. The cows were ca. 6 years old, and were fed mostly on grass and maize silage. The urine and faeces were stored for up to 2 days before removal from the cold room the night before application, to allow the urine/dung to attain ambient temperature before application.

The artificial urine was prepared following the recipe of (Kool et al., 2006a) and contained: urea (7.92 g N l^−1^), hippuric acid (0.53 g N l^−1^), allantoin (1.46 g N l^−1^), uric acid (0.08 g N l^−1^), creatinine (0.33 g N l^−1^); as well as KHCO_3_, KCl, CaCl_2_·2H_2_O, MgCl_2_·5H_2_O and Na_2_SO_4_. Both urine (natural and artificial) and dung were analysed for pH (ratio urine/dung to water 1:6); dry matter (DM) (drying at 105 °C ± 5 for at least 12 h); total C and total N by combustion of the sample followed by separation by a GC column and analysed by a TCD detector; readily available N (i.e. ammonium and nitrate) by the formation of a diazo compound between nitrite and sulphanilamide, which is then coupled with *N*-1-Napthylethylenediamine dihydrochloride to give a red azo dye (colour measured at 540 nm) (APHA, 1976). Total organic carbon (modified Walkley-Black) was analysed by acidification followed by oxidation of the carbon and further analyses by TOC analyser. Sub-samples of bulked fresh and artificial urine were also collected at application to determine urea, hippuric acid, allantoin, uric acid and creatinine content (Table 3). Briefly, sub-samples were diluted 1:3 with HPLC grade de-ionised water before addition of either 1 M sulphuric acid (to pH 3) or 9 μl chloroform to prevent sample degradation before analysis. Preserved samples were analysed using HPLC-UV (Phenomex Luna C 18 (2), 250 mm × 4.6 mm; pH 4, flow rate 1.0 ml min^−1^) with the diode ray detector set at 218 nm. The rate of application for urine was the equivalent of 5 L m^−2^; dung was applied at 20 kg m^−2^. These rates are in the range of real deposition during grazing (Sugimoto and Ball, 1989, de Klein et al., 2014). DCD was applied at a rate of 10 kg ha^−1^, equivalent to 6.5 kg N ha^−1^ (according to commercial guidelines, (Moir et al., 2007), and mixed with the urine prior to application. Further details are given in Table 2, including periods of measurements, harvest dates and N applied for each treatment.

### 2.3 Experimental design

Each experiment (spring, summer and autumn) was setup as a randomised block design, with three replicates per treatment. Plots dimensions were 6 m × 3 m, with areas for measurements of GHG emissions, soil mineral N and moisture, grass DM production and plant N uptake (see also Bell et al., 2015b).

On each plot, there were five urine patches and five dung pats each measuring 60 cm × 60 cm. Each urine patch received 1.8 l of urine (a typical volume for a cattle urination event e.g. Misselbrook et al., 2016), which was applied using a watering can that was fitted with a perforated spray head. The application area was bordered by a frame to prevent the urine running off the area during application. The frame was removed once the urine had soaked into the soil. Each dung patch received 4 kg of fresh dung, spread to an even thickness across the 60 × 60 cm area. On each plot, an additional 2 m × 2 m area was treated with dung or urine. This area was used for soil mineral N and grass DM production and N uptake measurements. For the NU + DCD treatment, the inhibitor was mixed with the urine prior to application to give a DCD application rate of 10 kg ha^−1^. DCD was mixed with the urine to maximise the uniformity of distribution of the very small amount of product over the treatment area and would simulate the effect of adding DCD to feed (Minet et al., 2016), water troughs (Welten et al., 2013) or rumen boluses (Ledgard et al., 2008).

### 2.4 Sampling strategies and measurements

#### 2.4.1 Greenhouse gases

Emissions of N_2_O and CH_4_ were measured for nearly a full year to fulfil IPCC recommendations for deriving EFs (IPCC, 1997, IPCC, 2000). Five static chambers of 40 cm × 40 cm × 25 cm height (inserted to a soil depth of 5 cm) were used on each replicate plot, to account for spatial variability. On each sampling occasion, 10 ambient air samples were taken as a surrogate for the chamber air sample at chamber closure (T0), and a gas sample was taken from each chamber 40 min after closure (T40) (Chadwick et al., 2014). For each block of the three experiments, one chamber was designated as a “linearity chamber” to check the linearity of the gas accumulation in the headspace by also taking samples at 20 and 60 min. The sampling was done between 10:00 and 12:00 as recommended by Bell et al. (2015a) and gas samples were placed in pre-evacuated 20 ml vials, transported to the laboratory and analysed within 2 days. N_2_O and CH_4_ were both measured with a Perkin Elmer Clarus 500 gas chromatograph fitted with a Turbomatrix 110 automated headspace sampler, an electron capture detector set at 300 °C for N_2_O analysis, and a flame ionization detector (FID) for CH_4_ analysis. Separation was achieved by a Perkin Elmer Elite-PLOT megabore capillary column, 30 m long and 0.53 mm i.d., maintained at 35 °C; N_2_ was used as carrier gas.

Sampling was more frequent in the period immediately after treatment application: 5 times per week for the first 2 weeks, which was then reduced to twice per week up to week 24, and then to monthly, totalling around 30 sampling occasions over the year. Daily means of the 5 chambers for each treatment/plot were calculated. Cumulative emissions using these daily means for each treatment and block were estimated using the trapezoidal integration method (Cardenas et al., 2010). When measurements were stopped only a few days short of a full year from the day of application, the last measured value was assumed to apply also for the last day of the year. Emission factors for N_2_O were calculated by subtracting the cumulative flux for the control treatment from the cumulative flux for each treatment and corresponding block. This difference was divided by the total N applied, resulting in three replicate EF values for each treatment.

#### 2.4.2 Soil measurements

Soil moisture content in each experimental block (to 10 cm depth) was determined on each gas sampling occasion from a bulked sample of three samples per block. On 15 occasions, samples were taken per plot (5 samples bulked per plot) for determination of soil mineral N, (NH_4_^+^ and NO_3_^−^) content. The soil moisture content was determined by a gravimetric method, and the mineral N determined using an autoanalyser (Skalar SANPLUS Segmented Flow Analyser; model 5000-02, Skalar (UK) Ltd., York, UK), following KCl extraction.

#### 2.4.3 Meteorological data

Daily rainfall and air and soil (5 cm depth) temperatures (min. and max.) were monitored for a full year after treatment application. Soil temperature (5 cm) was also measured using a digital thermometer (Fisher Scientific, UK) every time gas sampling was carried out to adjust gas concentrations to standard temperature for flux calculations.

#### 2.4.4 Grass measurements

The grass yield at harvest (once following autumn application, twice following spring and autumn applications) was taken from a 1 m × 1 m quadrat within the 2 m × 2 m treated patch and from the untreated control plots, and DM determined by drying at 85 °C for 24 h. The grass N content was determined on ground dried material, which was placed in a vial until analysis using a Carlo Erba NA2000 elemental analyser interfaced with a Sercon 20:22 isotope ratio mass spectrometer.

### 2.5 Statistical analysis

Statistical analysis was carried out using Genstat v. 14 (VSN International). Cumulative N_2_O fluxes had normal distributions (so no data transformation was needed). Meta-analysis was applied to the cumulative N_2_O fluxes, EFs, CH_4_ emissions and herbage yield and N content to assess the effect of treatments over the seasons for season and treatment main effects and the interaction. The controls were excluded from this analysis in order to compare the treatments only for the cumulative N_2_O. In order to assess differences between controls a randomised Block Design ANOVA was applied to the controls only. Associations between daily N_2_O fluxes and soil N, soil moisture and temperature, and rainfall were assessed for all treatments and season of application. As a result, we assessed the importance of these factors on N_2_O emissions and fitted a statistical model to the data to predict emissions.

## 3 Results

The N input from the NU treatment varied between 405 and 435 kg N ha^−1^, and for the AU between 423 and 481 kg N ha^−1^, with a higher N application in AU except for the autumn application. (Unfortunately variability in the making of the artificial urine resulted in this large variation in N application rate). Allantoin, hippuric acid and creatinine were higher in the AU than the NU applied (see Table 3). Uric acid was higher in the NU than in the AU but urea contents were similar. The total N applied in dung was higher than for the urine treatments, with the highest application rate in the spring, followed by the autumn and summer.

### 3.1 Weather

**Fig. 2:**
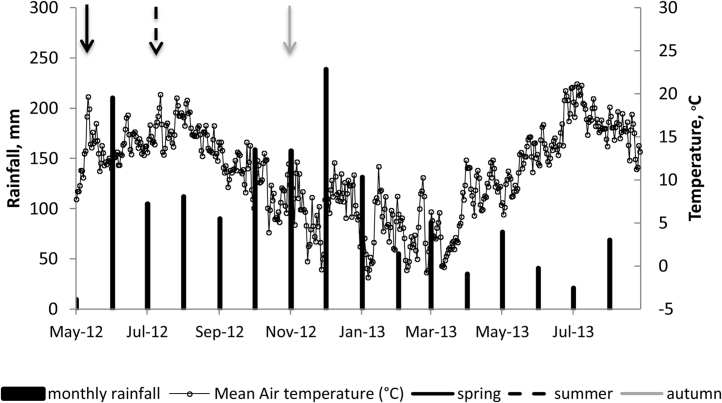
Mean daily air temperature and rainfall for Beacon Field. The arrows denote the time of urine and dung applications for each of the 3 experiments (15/5/12, 3/7/12 and 25/9/12 for spring, summer and autumn applications, respectively).

Rainfall was generally more frequent in 2012 than in 2013, with the latter year being also drier overall (Fig. 2). After February 2013, there were alternate periods of wet and dry weather (in terms of net differences between the long term average rainfall and the rainfall during the experiment), but mostly dry periods. Cumulative monthly rainfall during the experimental period was mostly greater (by up to 73%) than the site 30-year mean monthly values. Only in 2013 were there 4 months drier than the long- term mean: rainfall in February, April, June and July was 39, 46, 28 and 64% of the corresponding month of the long-term record, respectively. The total rainfall in the first 30 days after application of the treatments was 131.5, 102.7 and 162.8 mm for the experiments with spring, summer and autumn applications, respectively. Specifically, for each of the experiments, the total rainfall during the period when the majority of the emissions occurred (first 2–3 months after application) was 325 mm for the spring application, 462 mm for the summer, and 337 mm for the autumn application experiments. The cumulative rainfall for the duration of each experiment was 1405, 1246 and 1288 mm for spring, summer and autumn applications, respectively.

There was rainfall on the day of application in all experiments, but generally a relatively dry period followed in the experiments begun in the spring and summer, whereas there was a wet period at the start of the autumn experiment (see arrows in Fig. 2).

The air temperature was relatively low between November 2012 and March 2013, from below zero to about 12 °C. Maximum values were 21 °C in July and August. Values were similar to the long term record, with average values on the day of application of 8, 15 and 11 °C for the spring, summer and autumn experiments, respectively. The average temperature for the 2 months’ period after application was 13.9; 13.6 and 9.1 °C for the spring, summer and autumn experiments, respectively.

### 3.2 N_2_O emissions

#### 3.2.1 Linearity of the fluxes in the headspace

The increase in N_2_O gas concentrations in the headspace with time after chamber closure (determined in one chamber per block on each sampling occasion) was linear in almost all cases, with only 6 out of 99, 6 out of 96, and 1 out of 90 being non-linear sets for the spring, summer and autumn experiments, respectively. This confirms the robustness of this methodology as reported by Chadwick et al. (2014).

#### 3.2.2 Daily fluxes

**Fig. 3:**
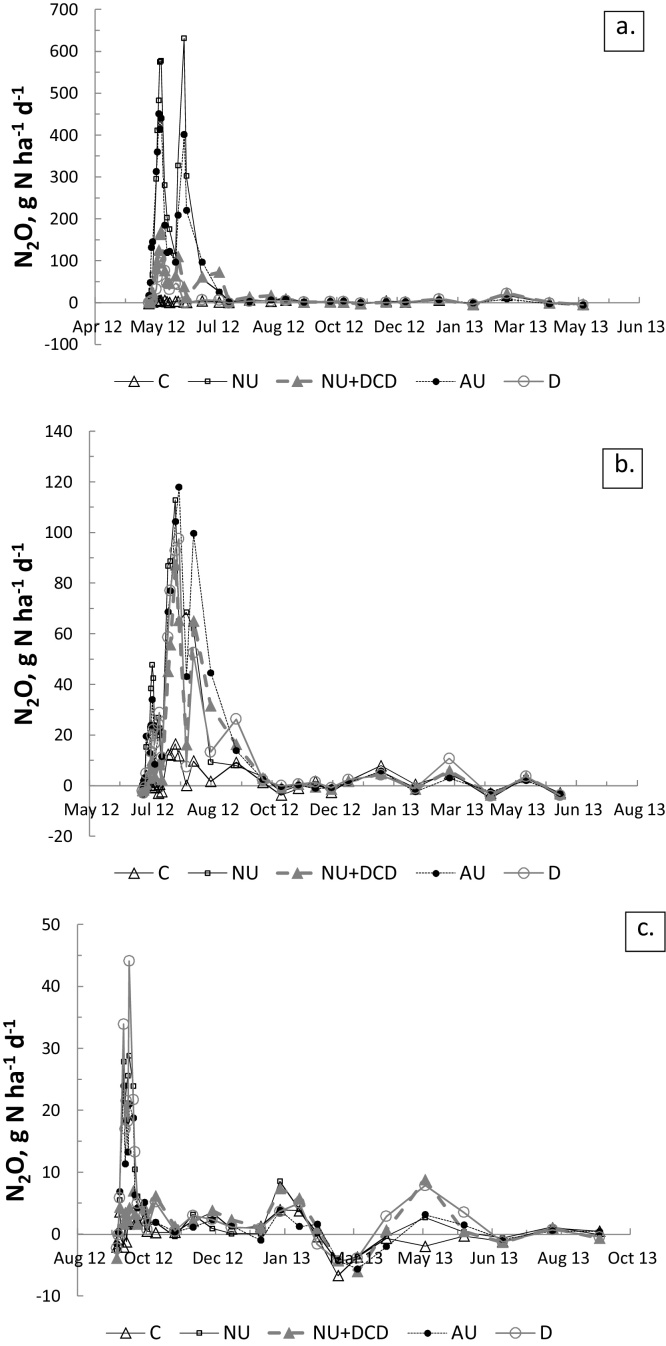
Daily N_2_O fluxes for the 2 experiments. a. Spring application; b. Summer application; c. Autumn application. (Note different y-axis scales).

Daily N_2_O fluxes after treatment application were largest in the spring experiment, with two clear peaks (the highest reaching 631 g N_2_O-N ha^−1^ d^−1^ for the NU treatment), followed by the summer and autumn experiments (Fig. 3). The NU + DCD treatment also showed a third peak later in the summer. The fluxes from the control were mostly zero.

Following summer application, multiple peaks were observed for all treatments, including the control. The largest flux, 118 g N_2_O-N ha^−1^ d^−1^, was from the AU treatment. Several peaks also appeared later in all treatments.

Following autumn application there were two small peaks for all treatments except for NU + DCD and C. The largest daily flux was from D, reaching 44 g N_2_O-N ha^−1^ d^−1^. There appeared to be measurable apparent negative fluxes in autumn 2013, reaching −7 g N_2_O-N ha^−1^ d^−1^.

#### 3.2.3 Cumulative emissions

There was a significant effect of application timing (season) on cumulative N_2_O emissions (Table 4) from the urine and dung treatments, with spring emissions higher than summer, and summer emissions higher than autumn for urine treatments. For D treatment: summer > spring > autumn. There was also significant effect of season × treatment interaction, but not from treatment only. For the controls the analysis showed that emissions were similar for spring and summer, but were significantly lower for autumn (P < 0.05). The results showed that DCD was effective in reducing emissions from the urine in the spring application (P < 0.001).

#### 3.2.4 Emission factors

The results of the meta-analysis (Table 4) showed that there was a significant effect of season (P < 0.001) and interaction season × treatment (P < 0.001). There was no significant effect of treatment (P > 0.05) on the EFs. Application timing (season) resulted in N_2_O EFs for the NU and AU treatments in the order spring > summer > autumn (Table 4, P < 0.001 in both cases). Values for the NU and AU treatments within seasons were similar only for autumn. The EF for the NU + DCD treatment was significantly lower following spring application than for the autumn and summer applications (P <0.001). For the D treatment, EF was greatest following the summer application (P < 0.001, Table 4).

For all treatments, there was a significant effect of season and season × treatment interaction on EFs. Only when removing AU from the dataset, treatment was an influential factor (P < 0.05). Removing the other treatments resulted in no significant change (P > 0.05 when removing D, NU and NU + DCD).

### 3.3 CH_4_ emissions

#### 3.3.1 Daily fluxes

Daily CH_4_ fluxes were larger following summer and autumn (data not shown), with larger peaks from the D treatment, especially in the autumn. Emissions from D appeared immediately after application in all experiments, with further peak events for the summer (up to 5 months after application) and autumn experiments (up to 8 months). Small positive fluxes occurred for all treatments for the summer and autumn experiments. Also, there were negative CH_4_ daily fluxes, especially in the spring and autumn experiments during the first few months with the lowest values −14.6, −4.9 and −6.7 g CH_4_ ha^−1^ d^−1^ for the spring, summer and autumn experiments, respectively.

#### 3.3.2 Cumulative CH_4_ emissions

**Fig. 4:**
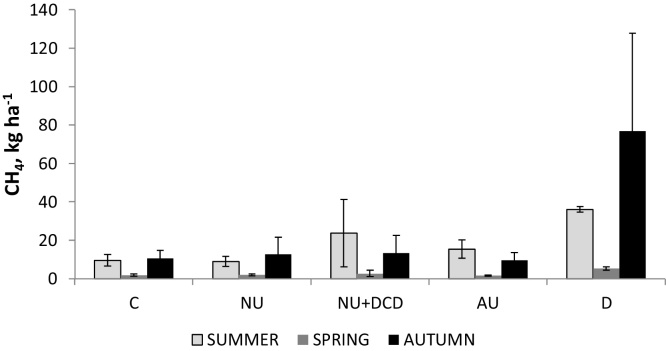
Cumulative CH_4_ emissions for the 3 experiments (bars are standard errors of the means).

Cumulative yearly emissions (Fig. 4 and Table 6) for all treatments ranged between 1.62 and 5.32 kg CH_4_ ha^−1^; 9.01 and 36.09 kg CH_4_ ha^−1^ and 9.61 and 51.05 kg CH_4_ ha^−1^ for spring, summer and autumn experiments, respectively. The meta-analysis showed that there was an effect of season (P < 0.002) but not of treatment (P = 0.052) or interaction season × treatment (see Table 6). Values were lowest following the spring application and generally greatest from the D treatment.

### 3.4 Soil water and N dynamics

**Fig. 5:**
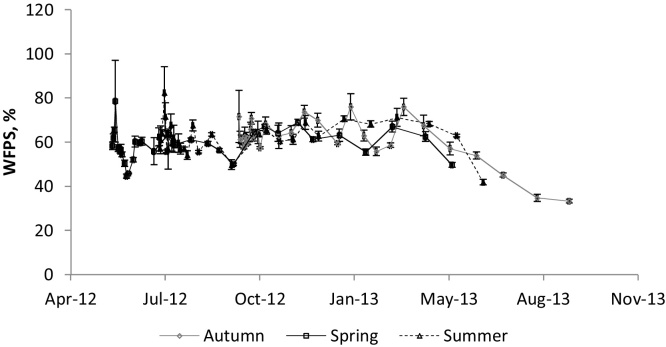
Soil moisture expressed as WFPS for the 3 experiments (bars are the standard error of the means).

The WFPS for the 3 experiments (Fig. 5) was relatively low, with annual means following the spring, summer and autumn applications of 59.1% (±6.9), 62.3% (±7.4) and 61.2% (±10.0), respectively (standard deviations in brackets). The lowest values were recorded in the autumn experiment in the last 2 months: down to 33%.

**Fig. 6:**
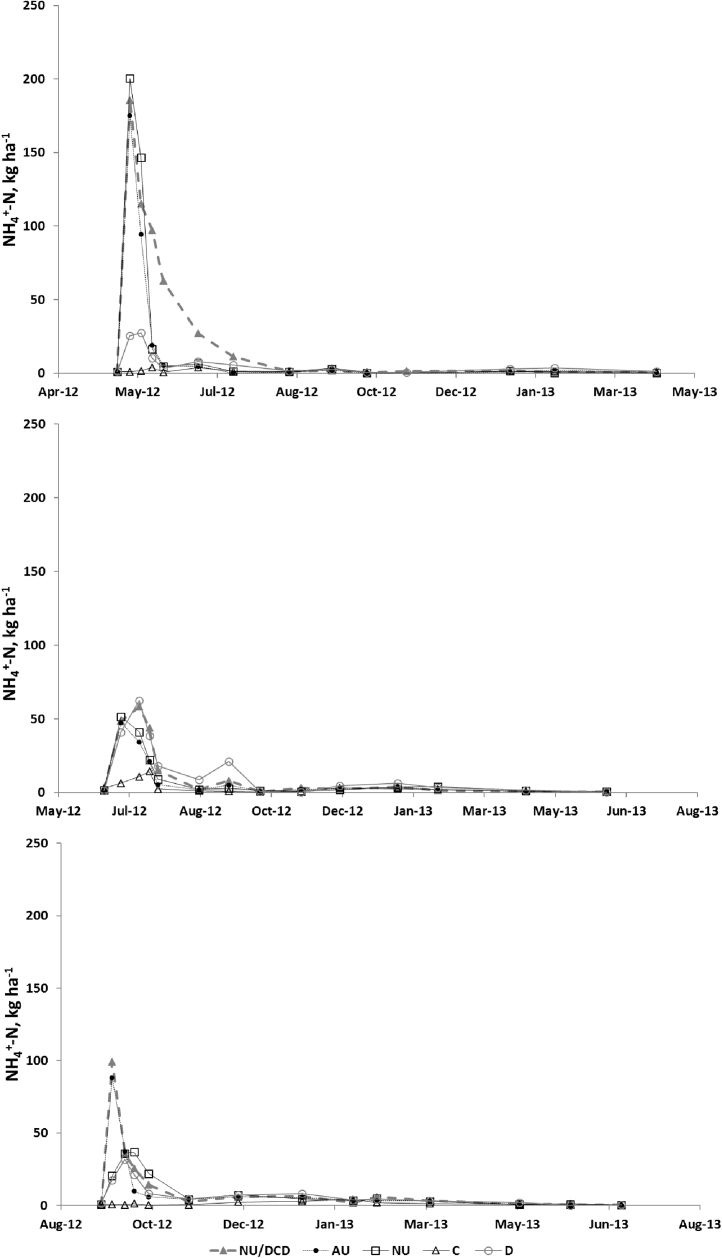
Soil NH_4_^+^-N for the a. spring, b. summer and c. autumn experiments.

Soil NH_4_^+^-N concentrations were largest following the spring application (Fig. 6). There was an increase in all treatments (except for the control) after application. Two peaks occurred following the summer application. Following spring application, concentrations were higher for the urine treatments, with no significant effect from DCD. In the autumn application, soil concentrations were higher when DCD was added with the urine.

**Fig. 7:**
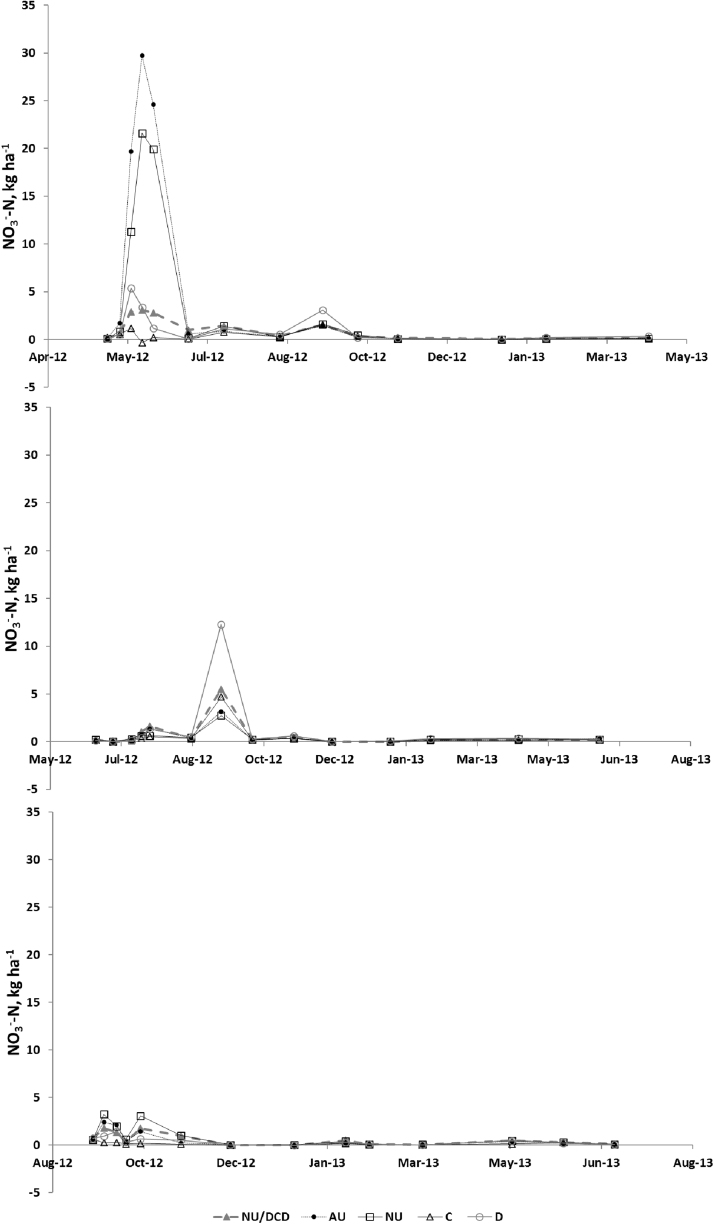
Soil NO_3_^−^-N for the a. spring, b. summer and c. autumn experiments.

Soil NO_3_^−^-N concentrations increased several weeks after treatment application (Fig. 7). There was a later increase in NO_3_^−^-N following the summer application. Two clear peaks appeared following the autumn application, with concentrations following the spring application being greater than for the summer and autumn applications. Application of DCD with urine seems to have decreased soil concentrations following the spring and autumn applications but not the summer.

Overall, soil mineral N was higher for the spring compared to the summer applications, which in turn were higher than for the autumn application.

The correlation analysis showed N_2_O emissions to be significantly correlated with soil NO_3_^−^, NH_4_^+^ and air temperature (62, 40 and 26%, P < 0.001) and; with soil moisture (−22%, P < 0.05). Soil moisture was negatively correlated with soil NO_3_^−^ (−33%, P < 0.001). Soil NO_3_^−^, NH_4_^+^ and air temperature were good predictors of emissions following the equation:

N_2_O emitted, g N ha^−1^ d^−1^ = −13.87 (7.74) + 7.694 (0.784) × Mean_soil_NO_3_^−^, kg N ha^−1^ + 0.5166 (0.0995) × Mean_soil_NH_4_^+^, kg N ha^−1^ + 1.369 (0.696) × Mean_air_temperature, °C; with a percentage variance accounting for 47.6% (values in brackets correspond to the standard errors of the coefficients).

### 3.5 Plant yield

Yields from control treatments in the first cut were similar in all three experiments: between 2.04 and 3.12 t DM ha^−1^ yr^−1^. The meta-analysis showed that for the first cut, treatment was significant. Season or the interaction season x treatment were significant. Values were not different for the urine treatments for each season, but were larger than C and D (Table 5). For the second cut, both season and treatment were significant but not their interaction. Larger values were for NU + DCD and D (but not significantly different between them) compared to the other urine treatments and the control (P < 0.001).

**Fig. 8:**
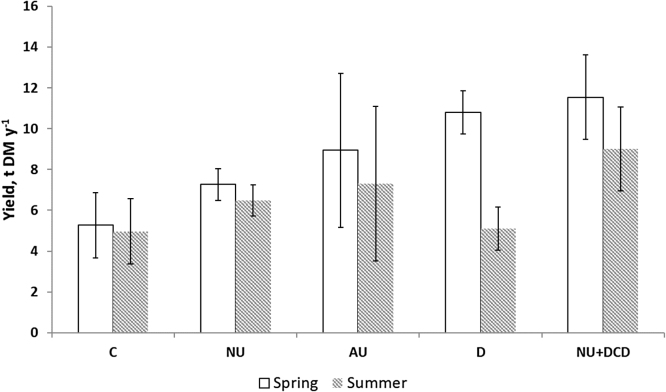
Annual yield for 2 cuts for all treatments for the spring and summer experiments (the autumn experiment is excluded from the graph as there was only one cut) (bars are standard errors of the means).

The total yields (2 cuts) were generally larger for spring than summer (there was not a second cut for the autumn experiment; see Fig. 8). Generally, the NU + DCD treatment gave the largest yields.

### 3.6 Plant N uptake

The meta-analysis showed that for the first cut, treatment and season but not the interaction season x treatment were significant. For the first cut, crop N offtake was smaller for the autumn application for all treatments. Values for the summer application were generally larger than for the spring, except for the dung treatment. For the second cut, crop N offtakes for the spring experiment were much larger than for the summer (there was no second cut for the autumn application) (Table 5). Generally, herbage yield and N offtake were larger for the NU + DCD treatment.

## 4 Discussion

Soil moisture (Fig. 5) expressed as% WFPS was not particularly high for the majority of the time (range was between 33.2 and 82.3%), possibly due to alternating periods of wet and dry weather, especially in the summer and autumn experiments. In the latter there was little rain after March 2013, explaining the very low WFPS values: ≤50%. However, it is possible that the weather on the day of application and prior to application might have influenced the responses observed. In the spring application, there was a little rain on the day of application (0.63 mm) and small amounts prior to this date, promoting mineralisation of the soil organic matter and producing available nitrogen, particularly nitrate; air temperature was high so this would have promoted microbial activity, particularly when water from the urine reached the soil, producing the large amounts of N_2_O observed.

For the summer experiment there were 7.56 mm of rainfall on the day of application and this was preceded by several days of similar volume. The fact that fluxes were smaller than in the spring may have been due to some N_2_O being reduced to N_2_.

In the autumn there were 11.13 mm on the day of application, preceded by large volumes of rain. It would be possible that there were losses of N via leaching explaining the lower fluxes observed.

### 4.1 Nitrous oxide emissions

Urine deposition is recognised as a potential source of hot spots for N_2_O emissions. The impact on fluxes is very much affected by environmental factors that modulate the microbial activity and transport of gases in soils. In addition to N input, urine deposition also increases soil moisture (Bol et al., 2004) which will also affect N_2_O emissions. The method of application of the urine in our experiments provided a urine patch that was larger than the chamber used for sampling gases. This means that we only measured the urine effect, not the effects caused by nearness to the edges of the patch. A genuine urine patch produced by a grazing cow can cover 0.16–0.49 m^2^ (Williams and Haynes, 1994) so to capture its full effect a chamber with a large surface area will be required, or several small chambers that will cover a transect with a radius longer than the patch’s. Marsden et al. (2016) estimated larger EFs (twice as large) when considering the diffusional area of the urine patch in a mesocosm study but their N rates were less than in our study (200 kg N ha^−1^) and only considered soil; no plant was included. The plant would have reduced this potentially higher EF by taking up some of the N that diffused horizontally.

The predominant WFPS values in all experiments were around 60%, the threshold from which denitrification becomes the main source process for N_2_O (Davidson, 1990). In addition, the greater part of the N in urine is as Urea (source of NH_4_^+^) so it is likely that when soil moisture is favourable (less than 60% WFPS), nitrification is an important source of N_2_O. This would suggest that nitrification could have had a predominant role as a source of emissions in our experiments. Similar results were shown by (Bol et al., 2004, Carter et al., 2006) in a Danish pasture. Carter et al. (2006) also attributed N_2_O emissions from nitrification after application of low N urine (0.7 g N l^−1^) to a sandy soil. They claimed that, under high N urine, nitrification will be inhibited for few days, possibly due to root scorching. We added about 10 times their N rate but did not have a delay in the response to the urine application in any of the three experiments, suggesting that scorching did not happen.

The occurrence of multiple N_2_O peaks after urine application has been observed before (Barneze et al., 2015) and also from fertiliser application by (Krol et al., 2016). These later peaks could be the result of newly formed available N after the initial application of the treatments (and appearance of the initial peak) and also due to the priming of the microbial activity. It could also be that the initial peak originated from nitrification of the NH_4_^+^ applied, and after treatment application, microbial respiration increased, exhausting O_2_ and promoting denitrification of the resulting NO_3_^−^. This has been reported by (Wrage et al., 2011) as nitrifying bacteria change to short-term denitrification via nitrifier-denitrification, especially when there are short periods of high moisture. Also, Marsden et al. (2016) suggested initial nitrification after urine application and possible onset of denitrification later on, generating further peaks of N_2_O. It is not clear in our case whether denitrification occurred, as soil moisture was relatively low, but there were short periods where moisture increased to 70–76% WFPS, and especially for the autumn experiment, when several peaks appeared 4 and 8 months after application. The opposite has been reported by Koester et al. (2015) where, after apportioning the pathway to N_2_O via an isotopomer approach, they proposed that initially denitrification dominated and was followed by nitrification once the carbon pool was exhausted. Our results differ from Boon et al. (2014), as in their case there was a delay in response after application of urine attributed to inactivity of nitrifier populations on swards that do not receive regular fertiliser. This was not the case in our experiments, as we had an immediate response after treatment application.

It is worth pointing out that the emissions resulting from both natural and artificial urine were similar in our study, unlike previous studies which have suggested that artificial urine may overestimate EFs (de Klein et al., 2003). This provides confidence that using artificial urine for research purposes (from Kool et al., 2006a) is a good alternative when natural urine is not available, or when manipulation of the urine composition is required.

#### 4.1.1 Effect of season on fluxes

The larger daily fluxes in the spring application are attributable to the addition of the N and C with the treatments, that stimulated microbial activity at a time of the year when temperatures started to increase. In the summer, large fluxes would have been expected due to higher temperature but this was not the case, possibly because a larger proportion of the N applied was lost as NH_3_. Ammonia volatilisation losses are typically low (<10% of urine N) following urine deposition to soil because of rapid infiltration, but substantially greater losses have been reported. For example, Laubach et al. (2013) in a study in New Zealand under summer conditions reported NH_3_ emissions of 25.5% of urine N and 11.6% of dung N. The smaller fluxes in the autumn experiment can be explained by the lower temperatures affecting microbial activity.

The inhibitory effect of DCD in spring suggests nitrification could have been the process responsible for emissions in these two experiments, but could also be that there was an indirect effect by inhibiting denitrification. The lack of effectiveness in the summer application could have been due to degradation of the inhibitor at the higher temperatures (McGeough et al., 2016). Emissions in the autumn were much lower and similar for all treatments, perhaps due to the lower temperatures resulting in less microbial activity which could explain the lack of effect of DCD.

In the NU and AU treatments, the N applied was generally larger in the summer and autumn application (up to 15%) compared to the spring (except for AU in the autumn experiment), so this does not explain the larger fluxes in the spring application.

#### 4.1.2 Effect of treatment and interactions between season-treatment

The results of the meta-analysis showing that there was no effect of treatment and season-treatment interaction in all treatments in the summer experiment, can be due to larger losses of N as NH_3_, affecting the amount of available N for nitrification/denitrification. The lack of effect in the autumn, can be due to the very small fluxes, so spatial variability could have overridden any differences between treatments. In addition, the lack of difference in fluxes between the NU and AU treatments indicates that the hypothesis suggesting that hippuric acid could act as an inhibitor of nitrification (van Groenigen et al., 2005a) is not true, at least for this soil. The amount of hippuric acid in our AU treatment was higher than in the NU (Table 3) in all three seasons (from 60% up to >1000%). It would be possible that the effect of urine and dung deposition lasts beyond the initial 3 months after application; however, the soil mineral N data showed later peaks (up to 5 months after application) in all treatments including the control. This suggests that these peaks are the results of soil mineralisation and climate, and not directly due to the treatments.

### 4.2 Emission factors in grazing grassland in the UK

The EFs obtained in our study for both natural and artificial urine were significantly different for the different experiments. Values fit well within previous findings when using cattle urine. Allen et al. (1996) reported a range of 0.0–2.3% for clay loam, sandy clay loam, sandy loam and peat soils. Their measurement period was no more than 140 days and the N rate applied was between 477 and 1190 kg N ha^−1^. van der Weerden et al. (2011) reported much lower EF values of 0.26–0.30% for a sandy loam with N rates of 496–551 kg N ha^−1^ over 125–173 days’ measurements. Other studies only covered up to 137 days and applied much higher N rates. However, in order to provide a single value, we have averaged results from the three experiments, resulting in 1.21 ± 1.53% for urine, and 0.21 ± 0.16% for dung. Assuming an average ratio of excreted N in urine and dung of 60%/40% (typical for the UK, see Webb, 2001), we derive a combined-excreta weighted average EF of 0.81%. This is two and half times less than the IPCC default value of 2%. The urine average is similar to values found in the literature, such as 0.9% from van Groenigen et al. (2005b) (mean of 10 studies). Bell et al., 2015a, Bell et al., 2015b reported an average EF from a similar study in Scotland of 1.1 and 0.2% for cattle urine and dung, respectively. The results from both the daily and cumulative emissions from the dung treatment showed generally lower values than urine in the spring experiment, probably due to lower availability of nitrogen in dung; this was also reflected in the lower EF. The similar values in the summer could have been influenced by NH_3_ volatilisation of the urine-N and dung-N, leaving less N for nitrification, whereas in the autumn it could have been due to less microbial activity at the lower temperature. DCD was efficient in reducing emissions from urine in the spring and summer by 63% and 12.5%, respectively. No significant effect in the autumn could be due to low microbial activity reflected in the low emissions measured. Bell et al., 2015a, Bell et al., 2015b reported no significant effect of DCD used with urine on N_2_O emissions in any season, although they did report a non-significant reduction in the urine EF for the spring application from 0.2 to 0.06% of applied N with the addition of DCD. Misselbrook et al. (2014) reported a 70% reduction in the EF for cattle urine with the inclusion of DCD from six experiments conducted in England, with mean EFs of 0.41 and 0.12% for urine and urine + DCD, respectively.

### 4.3 N dynamics

The high initial NH_4_^+^-N in the soils in all treatments except for C and D, was provided by the urine applied. The larger values for the spring application compared to summer for AU, NU and NU + DCD could have been due to greater N losses as NH_3_ in the summer, as temperatures were higher. The higher values for AU and NU treatments in the autumn compared to the summer experiment are compatible with reduced loss of applied N in the cooler temperatures experienced in the autumn at the time of application. This effect is likely to be less important in the dung treatment, as NH_4_^+^-N contents are relatively low.

The soil NO_3_^−^ peak in the spring experiment lasted several weeks after the application of the treatments, probably due to on-going nitrification of the added NH_4_^+^. The delay in the summer peak could have been due to less NH_4_^+^ being available, due to volatilisation of NH_3_ and/or losses of NO_3_^−^ from denitrification, especially as soil moisture was relatively high. There was (data not shown) a decrease in NH_4_^+^ associated with the increase in N_2_O, even though N_2_O is relatively very small compared to NH_4_^+^ (by 3 orders of magnitude). The NO_3_^−^ data show very different behaviour for the three experiments in relation to the N_2_O peak. This could be due to the soil moisture status and effect of temperature. The correlation analysis showed a large influence of both soil NH_4_^+^ and NO_3_^−^ on N_2_O emissions, as well as air temperature and soil moisture. Generally, it seems nitrification prevailed in this study as a source of N_2_O, particularly influenced by the low WFPS during the whole period, but some denitrification could have occurred due to the influence of soil NO_3_^−^.

### 4.4 Plant yields and N uptake

The results from the yield data were generally consistent with more efficient uptake of nutrients in the early seasons as expected due to plant growth. DCD was effective in increasing yield and N in the plant in the summer experiment, coinciding with the least effect on N_2_O emissions. The increase in yield and herbage N has been observed before (Di and Cameron, 2006).

### 4.5 Methane emissions

Fluxes of CH_4_ are not normally expected to occur from soils under the circumstances of our experiment, when soil moisture was low. However, some large positive fluxes were observed from the dung treatment, possibly due to anaerobicity promoted by the dung lying on the soil surface after application. Measurements of CH_4_ have been reported from cattle dung and urine patches in England (Yamulki et al., 1999), with an average of 57 and 0.2 mg CH_4_ per dung (1.2 kg) and urine (200 ml) patch (20 cm diameter), respectively. A review by Saggar et al. (2004) also reports positive fluxes from dung patches immediately after deposition. Methane uptake was reported in urine and dung patches from grazing sheep in Mongolia (Jiang et al., 2012), resulting in −0.076 and −0.084 g m^−2^ in a 100 days experiment. Flessa et al. (1996) reports values of 84.5 g CH_4_-C ha^−1^ (or 112.67 g CH_4_ ha^−1^) for a period of 78 days, during which there was one week of grazing; much smaller than our reported values. Jarvis et al. (1995) reported values of 17,020 g CH_4_ yr^−1^ for 10 days after deposition in SW England, in the middle of the ranges for the summer and autumn experiments of our study. This shows that there is a large potential for underestimating fluxes when measurements don’t cover the full year. The cumulative values from our study showing no effect of treatment suggest that at the low soil water contents during the 3 experiments microbes were stimulated regardless of the treatment applied.

## 5 Conclusions

N_2_O emissions mostly occurred within the first 3 months after application of urine in each experiment with cumulative emissions decreasing in the order spring > summer > autumn. EF values were 2.96, 0.56 and 0.11% of applied N for urine for spring, summer and autumn applications, respectively. The N_2_O EFs for dung were much smaller than for urine when added in spring and autumn with values of 0.14, 0.39 and 0.10% for spring, summer and autumn applications, respectively. The combined EF for excreta resulted in 0.81% for urine and dung at a ratio of 60%/40%. We found that the nitrification inhibitor, dicyandiamide, DCD, was effective in reducing N_2_O emissions for the spring application only. There were significant differences in N_2_O EFs between treatments in the spring (largest from urine and lowest from dung) but not in the summer and autumn applications. We also found that there was a significant effect of season (largest in spring) but not of treatment on the N_2_O EFs.

Hot spots of urine (and dung) need to be considered as affecting an area that will depend on soil conditions (moisture and texture) as well as their lasting effect due to initiation of microbial processes underpinning N transformations.

Methane emissions were larger and not significantly different from the dung application but there were no significant differences between treatments across season of application. Smallest cumulative values were in the spring for all treatments.

The occurrence of multiple N_2_O peaks is a subject that needs further research, using tools such as isotopes and isotopomers, and microbial measurements, particularly under different weather patterns which are directly affecting soil N transformation and changes in fluxes. They could be the result of different processes occurring due to changes in aeration status. Large differences in soil N mass and N_2_O fluxes of several orders of magnitude do not seem to override these effects.

## Figures and Tables

**Table 1:** Soil characteristics (standard error in brackets).

Site	North Wyke
Field name	Beacon Field
Soil type (soil series)	Clay
pH	5.73
Olsen P, extractable (ppm)	28.3
K extractable (ppm)	197.3
Mg extractable (ppm)	102.7
Org C (%)	5.37
Total N (%w/w)	0.52
BD (g cm^−3^)	0.62 (0.01)
Sand, 2.00–0.063 mm	13.6 (5.6)
Silt, 0.063–0.002 mm	43.2 (3.0)
Clay, <0.002 mm	43.2 (6.4)

**Table 2:** Description of the three experiments.

	Spring	Summer	Autumn
Start date	15/05/2012	03/07/2012	26/09/2012
End date	09/05/2013	11/06/2013	10/09/2013
Natural urine N loading (kg N ha^−1^)	405	429	435
Artificial urine N loading (kg N ha^−1^)	440	481	423
Dung N loading (kg N ha^−1^)	911	625	771
Natural urine + DCD N loading (kg N ha^−1^)	395	436	454
Harvest dates	19/6/2012, 28/08/2012	09/08/2012, 25/05/2013	25/5/2013

The N in the DCD is taken into account in the total N applied for the NU + DCD treatment.

**Table 3:** Urine and dung composition for all three experiments (nd = not determined).

Experiment	Application	DM	Total N	NO_3_^−^	NH_4_^+^	pH	TOC	LOI	Compound in urine				
									allantoin	creatinine	uric acid	hippuric acid	urea
		g l^−1^	g l^−1^	mg l^−1^	mg l^−1^		mg l^−1^	g 100 g DM^−1^	mg l^−1^	mg l^−1^	mg l^−1^	mg l^−1^	mg N l^−1^
Spring	Natural urine	53.3	8.1	<0.1	554	8.25	14944	nd	1905.6	761.7	368.0	3923.7	6521.4
	Artificial urine	43.0	8.8	<0.1	17.6	8.22	9238	nd	4306.4	920.8	221.1	6606.9	7078.6
	Natural urine + DCD	52.3	7.9	<0.1	644	8.26	14725	nd	nd	nd	nd	nd	nd

Summer	Natural urine	48.2	8.57	1.04	1230	7.33	nd	55.2	<400	520	396	<500	6284
	Artificial urine	41.9	9.61	0.43	<50	7.51	nd	28.9	3031	768	68	6120	6833
	Natural urine + DCD	47.1	8.72	0.84	1090	7.28	nd	55.8	nd	nd	nd	nd	nd

Autumn	Natural urine	45.0	8.70	2.51	2020	9.17	nd	45.2	<400	519	347	4859	7382
	Artificial urine	34.4	8.45	0.75	<50	7.42	nd	41.6	3632	732	219	6008	7774
	Natural urine + DCD	40.8	9.07	2.29	2840	9.09	nd	45.7	nd	nd	nd	nd	nd

Experiment	Application	DM	Total N	NO_3_^−^	NH_4_^+^	pH	TOC	LOI
		g kg^−1^	g 100 g DM^−1^	mg kg DM^−1^	g 100 g DM^−1^		mg L^−1^	g 100 g DM^−1^
Spring	Dung	145	3.14	<0.1	0.304	7.02	211150	nd
Summer	Dung	211	1.48	21.6	0.431	7.44	nd	25.1
Autumn	Dung	205	1.88	20.8	0.294	7.46	nd	46.8

**Table 4:** Cumulative N_2_O emissions and EF means (calculated from the meta-analysis) N = 3.

Treatment	NU	AU	NU + DCD	D	C
Total N_2_O spring (g N ha^−1^)	13257.9^Dc^	11058.6^Cc^	5544.7^Bc^	2500.5^Ac^	1256.1^b^
Total N_2_O summer (g N ha^−1^)	3191.6^Ab^	4163.9^Bb^	2929.2^Ab^	3244.2^Ab^	804.1^b^
Total N_2_O autumn (g N ha^−1^)	517.8^Aa^	337.3^Aa^	586.4^Aa^	824.9^Aa^	31.5^a^
EF Spring (%)	2.96^Dc^	2.23^Cca^	1.09^Bc^	0.14^Aa^	n/a
EF summer (%)	0.56^Bb^	0.70^Cb^	0.49^Ab^	0.39^Ab^	n/a
EF autumn (%)	0.11^Aa^	0.072^Aa^	0.12^Aa^	0.10^Aa^	n/a
Probability (EF)	Treatment: 0.293; Season:<0.001; Interaction: <0.001				
Probability (Total N_2_O)	Treatment: 0.145; Season:<0.001; Interaction: <0.001 For the controls: Season: 0.021; SED: 163.8				

From the metaanalysis: **For EF means**, the Standard errors of differences (SED) were: Treatment Means within each Season = 0.1334; Season Means within each Treatment = 0.1506 and Interaction Means = 0.1459.

**For cumulative N_2_O**: The Standard errors of differences (SED) were: Treatment Means within each Season = 551; Season Means within each Treatment = 678.9 and Interaction Means = 644.

Values in parentheses for the control are the standard errors.

Superscripts are the significance of the differences in treatments, in upper case representing the comparison between treatments for each season (between rows); the lower case represents the comparison between seasons for each treatment (between columns).

The metaanalysis for the total N_2_O was carried out excluding the controls, so the standard errors in parenthesis correspond to the experimental values.

If interaction is significant then compare treatment within season and season within treatments. If interaction not significant, take the marginal treatment means and marginal season means.

**Table 5:** Grass yields and N offtake for all treatments and season applications N = 3.

	First cut (t DM ha^−1^)			Second cut (t DM ha^−1^)^a^	
	spring	summer	autumn	spring	summer
C	2.04^A^	3.12^A^	2.15^A^	3.23^A^	1.85^A^
NU	3.45^B^	4.64^B^	4.21^B^	3.81^AB^	1.85^AB^
AU	3.87^B^	5.62^B^	3.55^B^	5.08^ABC^	1.68^ABC^
NU + DCD	3.17^B^	6.88^B^	3.24^B^	8.37^BC^	2.13^ABC^
D	3.16^A^	2.04^A^	3.00^A^	7.63^C^	3.06^C^
Probability first cut	Treatment: 0.013; Season: 0.054; Interaction: 0.277				
Probability second cut				Treatment: 0.021; Season: P < 0.001; Interaction: 0.222	

	First cut (kg N ha^−1^)			Second cut (kg N ha^−1^)	
	spring	summer	autumn	spring	summer
C	42.01^Ba^	84.38^Ba^	31.15^Aa^	69.10^a^	34.04^a^
NU	144.25^Bbc^	160.54^Bbc^	71.16^Abc^	97.70^ab^	33.97^ab^
AU	161.40^Bbc^	188.25^Bbc^	57.29^Aabc^	124.23^abc^	30.44^abc^
NU + DCD	143.64^Bc^	241.70^Bc^	54.25^Ac^	247.75^c^	37.41^c^
D	129.81^Bab^	77.69^Bab^	52.64^Aab^	218.65^bc^	58.04^bc^
Probability (First cut)	Treatment: 0.011; Season: P < 0.01; Interaction: 0.120				
Probability (Second cut)				Treatment: 0.006; Season: P < 0.01; Interaction: 0.115	

From the metaanalysis: **For First cut dry matter**, the Standard errors of differences (SED) were: Treatment Means within each Season = 1.073; Season Means within each Treatment = 1.096 and Interaction Means = 1.089.

**For First cut N**, the Standard errors of differences (SED) were: Treatment Means within each Season = 36.06; Season Means within each Treatment = 39.75 and Interaction Means = 38.70.

Superscripts are the significance of the differences in treatments, in upper case representing the comparison between treatments for each season (between rows); the lower case represents the comparison between seasons for each treatment (between columns).

a For the second cut there are only 2 seasons, as the interaction was not significant we assessed the treatment means only.

**Table 6:** Cumulative mean CH_4_ fluxes (kg CH_4_ ha^−1^). N = 3.

Treatment	Spring	Summer	Autumn
C	1.898^A^	9.619^B^	10.637^A^
NU	2.047^A^	9.011^B^	12.723^A^
NU + DCD	2.720^A^	23.801^B^	13.447^A^
AU	1.624^A^	15.435^B^	9.611^A^
D	5.317^A^	36.089^B^	76.835^A^
Probability (CH_4_)	Treatment: 0.052; Season: 0.002; Interaction: 0.250		

From the metaanalysis: The CH_4_ mean values: Standard errors of differences (SED) were: Treatment Means within each Season = 8.523
